# Smokeless tobacco: knowledge, attitudes and usage in Pakistan

**DOI:** 10.1186/s41043-025-00754-0

**Published:** 2025-01-24

**Authors:** Hammad Atif Irshad, Sajjan Raja, Hamzah Jehanzeb, Wamiq Ali Shaikh, Umair Saleem, Shahzil Abdur Rehman Malik, Akmal Shahzad, Mamoon Shaikh, Nousheen Iqbal, Javaid Ahmed Khan

**Affiliations:** 1 Medical College, Aga Khan University, Karachi, 74800 Pakistan; 2 Ziauddin University, Karachi, Pakistan; 3 Jinnah Medical and Dental College, Karachi, 74800 Pakistan; 4 Section of Pulmonology and Critical Care, Department of Medicine, Aga Khan University Hospital, Karachi, 74800 Pakistan

**Keywords:** Chewed tobacco, Public health, Snus, Non-cigarette, Smokeless tobacco

## Abstract

**Background:**

Smokeless tobacco (SLT) encompasses products that are not burnt but instead consumed orally or nasally. One-third of tobacco is consumed in the smokeless form in South Asia. Despite its widespread usage, there has been limited empirical research on the prevalence and factors influencing SLT consumption in Pakistan. This study aimed to provide an assessment of SLT knowledge, attitudes, and usage in Pakistan.

**Methods:**

Using an online questionnaire, a cross-sectional survey was conducted across Pakistan. Convenience sampling was used to disseminate, and expert approval was sought followed by a pilot study to validate the questionnaire. A comprehensive questionnaire was developed using elements from the Global Adult Tobacco Survey and other tools which had previously been utilized within Pakistan. Participants’ responses were described using descriptive statistics, and SPSS version 26 was used to perform linear and logistic regression. A p-value of less than 0.05 was considered statistically significant.

**Results:**

Data was collected from 1,195 participants among which 85.86% of participants had heard of SLTs prior to the survey. Sex, province, household income and previous doctor visits were significant determinants to product knowledge through which 72.55% agreed that these products can lead to serious illnesses but 30% noted a lack of knowledge on legality. General attitudes agreed that it makes one feel unwell (44.1%), gives a sense of guilt (43.85%) and more research is needed on it (54.39%). Negative health effects were the biggest deterrent from pursuing these products for 60.33% of participants. Among significant factors influencing the use of SLTs, the greatest odds of use were found with use as an alternative to cigarettes (OR 4.45) and secondly, due to its availability in a liked flavor (OR 2.27). About 31% reported to have used the product once, with 69.82% of current users expressing a desire to quit.

**Conclusions:**

Although adequate knowledge and aligning attitudes exist for SLTs, light is shed in the face of a sustaining public health problem. We offer important insights that can guide the creation of focused interventions meant to lower SLT use, and the health hazards associated with it in the Pakistani community by highlighting important myths, incentives, and deterrents.

## Introduction

From a vast range of methods used across the globe, tobacco use can be summarized into the two major categories of smoked and smokeless tobacco (SLT) products [1]. SLTs encompass those products that are not burnt but instead consumed orally or nasally [2]. These include moist snuff (held between the cheek and gum; available in various forms such as Khaini, Snus, Shammaah, Nass, or Naswar), dry snuff (powdered tobacco traditionally inhaled through the nose or taken orally) and chewing tobacco (placed in the mouth and either sucked or chewed [1]. Varieties of SLT include Plug, Loose-leaf, Chimo, Toombak, Gutkha, Twist and Pan-masala or Betel-quid, which comprises tobacco, areca nuts, slaked lime, sweeteners, and flavorings wrapped in a betel leaf [1].

Despite not being combusted, SLTs contain harmful substances including at least 28 cancer causing chemicals (carcinogens) that can lead to numerous health issues [3, 4]. These products pose significant risks to oral health, including gum disease, tooth decay, and oral cancer, stemming from prolonged exposure to carcinogens and irritants [3]. Furthermore, SLT increases the risk of various cancers beyond the oral cavity, such as esophageal and pancreatic cancer, while also fostering nicotine addiction, complicating cessation efforts. Cardiovascular consequences, including elevated heart rate and blood pressure, contribute to the potential for cardiovascular disease, while reproductive health may also suffer, with implications for fertility and pregnancy outcomes [3, 5, 6].

The prevalence of SLTs is influenced by a variety of demographic factors, including socioeconomic status, ethnic background, gender, and age [3, 5]. These products are most used in South Asian countries, where one-third of tobacco is consumed in the smokeless form [7, 8]. Increased use of smokeless tobacco products is highly neglected as international governments are more focused on eliminating cigarettes and not tobacco entirely, which is contrary to article 1 (d) & 1(f) of WHO’s Framework Convention on Tobacco Control [9].

Furthermore, there is no regulation on the manufacturing and sales of SLTs in low- and middle-income countries like Pakistan. This leads to and supports purchase of SLTs, while misconceptions of it being less hazardous or even having medical benefits prevail [10, 11].

From a public health point of view, the use of SLTs poses a massive challenge globally. Contrary to popular belief that smokeless tobacco provides an avenue for current cigarette smokers to quit, it was found that the initial use of SLTs instead paved the way for many adolescents to move on to cigarette smoking [12]. The use of SLTs is generally considered socially acceptable and there is a lack of knowledge regarding its detrimental effects on health. In fact, it has been observed that the misconception about the favorable effect of chewing SLTs is common, primarily among the younger age groups [13, 14].

In Pakistan, SLT is predominantly consumed in various forms such as Naswar (tobacco mixed with cardamom and menthol) and Paan or Betel-quid [2]. Despite its widespread usage, there has been limited empirical research on the prevalence and factors influencing smokeless tobacco consumption in Pakistan [2]. Studies examining the correlation between the use of SLT and other substances like areca nut, and their association with head, neck, and oral cancers, have been conducted in specific regions of Pakistan, often with small sample sizes [15–21].

This study aimed to provide a comprehensive examination of SLT consumption in Pakistan, offering insights into its knowledge, attitudes, motivations, deterrents, and usage patterns among the general public.

## Methods

The Strengthening the Reporting of Observational Studies in Epidemiology (STROBE) Statement’s reporting guidelines are adhered to in this cross-sectional study [22].

### Study design, setting and population

This cross-sectional study was approved by the Ethical Review Committee (ERC) (ID: 2023-8608-24823) in Aga Khan University Hospital (AKUH), Karachi, Pakistan and was carried out throughout Pakistan using an online questionnaire.

#### Inclusion criteria

Adult (age 18 or older) residing in Pakistan who provide consent to participate in the study and understand and can respond in Urdu or English.

#### Exclusion criteria

Below 18 years of age, not residing in Pakistan and unable to respond to questions in Urdu or English.

### Sample size calculation and sampling technique

The sample size was calculated using OpenEpi [23]. According to the Pakistan Demographic and Health Survey 2017-18, the current smoking prevalence among men ages 15–49 is 31.8% [24]. Using that statistic, at least 334 participants aged 18 years and above were determined to be recruited. An additional 10% margin of error was taken for a minimum required sample size of 367.

For this study, convenience sampling was used to reach adults across the country. The online questionnaire was shared on social media platforms and circulated from person to person.

### Data collection tool

In the absence of a prior questionnaire suitable for our population, a comprehensive questionnaire was developed using elements from the Global Adult Tobacco Survey (GATs) and other tools which have previously been utilized within Pakistan [14, 25–27].

The English questionnaire was also translated to Urdu, which is the national language of Pakistan, by an independent translator fluent in both languages and with experience in questionnaire translation.

To ensure face validity, the English and Urdu versions of the questionnaire underwent pilot testing among 50 respondents. A 7-item knowledge, 27- item attitude and 5 item usage questionnaire were used for pilot testing. Cronbach’s alpha value was calculated for each of these domains to check for internal consistency, with the calculated values being 0.8597, 0.9503 and 0.86 for the knowledge, attitudes, and usage domains respectively. These scores reflected good, excellent, and good consistency respectively.

The final components of the questionnaire were then rigorously reviewed in close association with faculty experts in tobacco research at the Section of Pulmonary and Critical Care Medicine at AKUH. Content validity was assessed by calculating a content validity index (CVI) for this survey for relevance, essentiality and clarity based on the ratings of two subject experts. A CVI of 0.942, 0.942 and 0.8986 were calculated for these three parameters respectively, which was deemed acceptable according to Davis (1992) [28].

The final survey contained four sections which were demographics, knowledge, attitudes, and usage. The survey began with a consent form explaining the nature and scope of the survey and then the sections were asked from the respondents.

### Data collection procedure

Responses were collected by a questionnaire that was available in both English and Urdu, the national language of Pakistan. Our questionnaire was circulated among adults (age ≥ 18) as a Google Form, on closed social media platforms, such as WhatsApp groups and Facebook groups.

As the data was collected, it was compiled into a Google Sheet. This Google Sheet was protected by a password and accessible only to members of the research team. Upon completion of data collection, Google Sheet was downloaded as an Excel sheet, which was password protected. The Google Sheet and individual questionnaire responses were available to the research team for 3 months after data collection was complete, after which they were erased.

### Statistical analysis

Statistical analyses were run using Statistical Package for Social Sciences (SPSS) version 26. Continuous data such as age were reported using mean and standard deviation. Categorical data consisting of the results of the Likert scales and multiple-choice questions has been reported as frequencies (gross numbers) and percentages (n; %). To analyze the effect of factors such as age, sex, and level of education on the overall knowledge score, a linear regression was performed. In addition, a logistic regression analysis was undertaken to determine significant motivating and deterring factors. A p-value of < 0.05 was considered significant for all analyses.

## Results

### Demographics

**Fig. 1:**
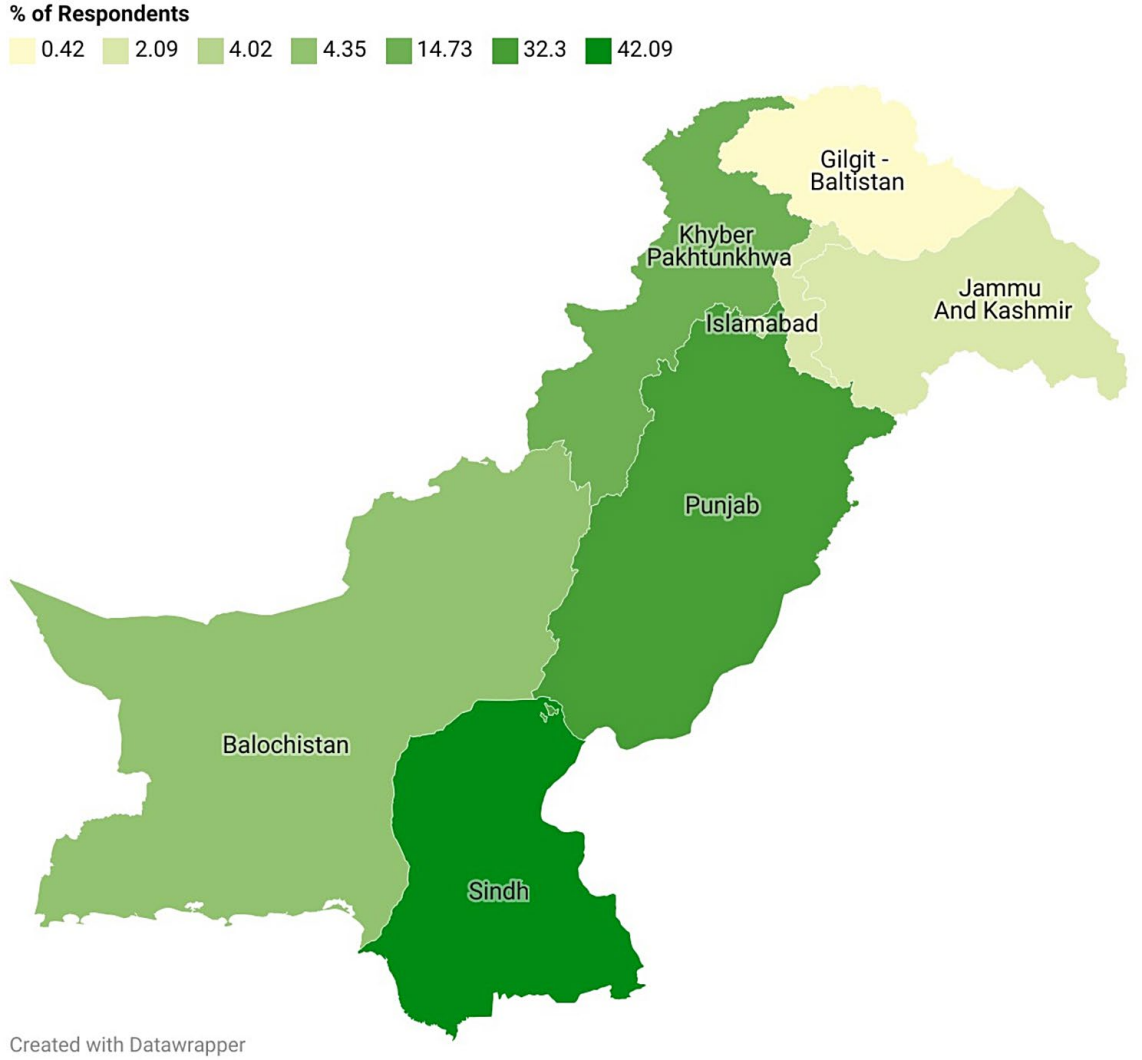
Geographical distribution of respondents

We collected data from a total of 1,195 participants. The median age of the participants stood at 23 years, and there was a fair distribution of gender, with 41.75% identifying as male and 58.24% as female. Geographically, the majority hailed from Sindh (42.09%) and Punjab (32.30%), while other provinces were represented to a lesser degree as displayed in Fig. 1. Ethnically, the largest contingent identified as Urdu speaking (29.29%), followed by Punjabi (22.51%), and Pashtun (17.49%). Educational levels varied, with 62.59% holding bachelor’s degrees and 26.11% possessing postgraduate degrees. Most of the respondents were unmarried (79.83%), while monthly income majorly ranged from PKR 100,000 to 500,000 (43.26%) and PKR 50,000 to 100,000 (26.69%). Additionally, the majority reported visiting a healthcare provider 1–2 times in the past 12 months (50.63%). These data have been summarized in Table 1.

**Table 1:** Demographics characteristics. *IQR: interquartile range;* *N = 1195.* *IQR has been reported as (Q1 – Q3)

Variable	*n* (%)/Median (IQR)*
**Age (Years)**	23 (5)
**Sex**	
Male	499 (41.75)
Female	696 (58.24)
**Province of residence**	
Sindh	503 (42.09)
Punjab	386 (32.30)
Khyber Pakhtunkhwa	176 (14.73)
Balochistan	52 (4.35)
Islamabad Capital Territory	48 (4.02)
Azad Jammu and Kashmir	25 (2.09)
Gilgit Baltistan	5 (0.42)
**City of residence**	
Karachi	434 (36.32)
Lahore	130 (10.88)
Peshawar	128 (10.71)
Bahawalpur	65 (5.44)
Islamabad	57 (4.77)
Quetta	51 (4.27)
Rawalpindi	46 (3.85)
Hyderabad	33 (2.76)
Multan	23 (1.92)
Gujrat	18 (1.51)
Tando Allahyar	13 (1.09)
Faisalabad	11 (0.92)
Rahim Yar Khan	10 (0.84)
Gujranwala	9 (0.75)
Muzaffarabad	9 (0.75)
Ghotki	8 (0.67)
Abbottabad	7 (0.59)
Charsadda	7 (0.59)
Narowal	7 (0.59)
Dera Ismail Khan	5 (0.42)
Mirpur Khas	5 (0.42)
Other	119 (9.96)
**Ethnicity**	
Punjabi	269 (22.51)
Urdu-speaking	350 (29.29)
Sindhi	156 (13.05)
Pashtun	209 (17.49)
Baloch	19 (1.59)
Mixed	52 (4.35)
Other	140 (11.72)
**Level of Education**	
< 5 years (Primary school)	2 (0.17)
5–10 years (Matric/ O Level)	9 (0.75)
10–12 years (Intermediate/ A Level)	124 (10.38)
12–14 years (Bachelor’s degree)	748 (62.59)
> 14 years (Postgraduate degree)	312 (26.11)
**Marital Status**	
Unmarried	954 (79.83)
Married	241 (20.17)
**Monthly Family Income (PKR)**	
< 25,000	69 (5.77)
25,000–50,000	118 (9.87)
50,000-100,000	319 (26.69)
100,000-500,000	517 (43.26)
> 500,000	172 (14.39)
**How many times did you visit a doctor or health care provider in the past 12 months?**	
0	
1 or 2	272 (22.76)
3 to 5	605 (50.63)
6 or more	218 (18.24)
	100 (8.37)

PKR (Pakistani Rupees); IQR (Interquartile range)

### Knowledge towards SLTs

**Fig. 2:**
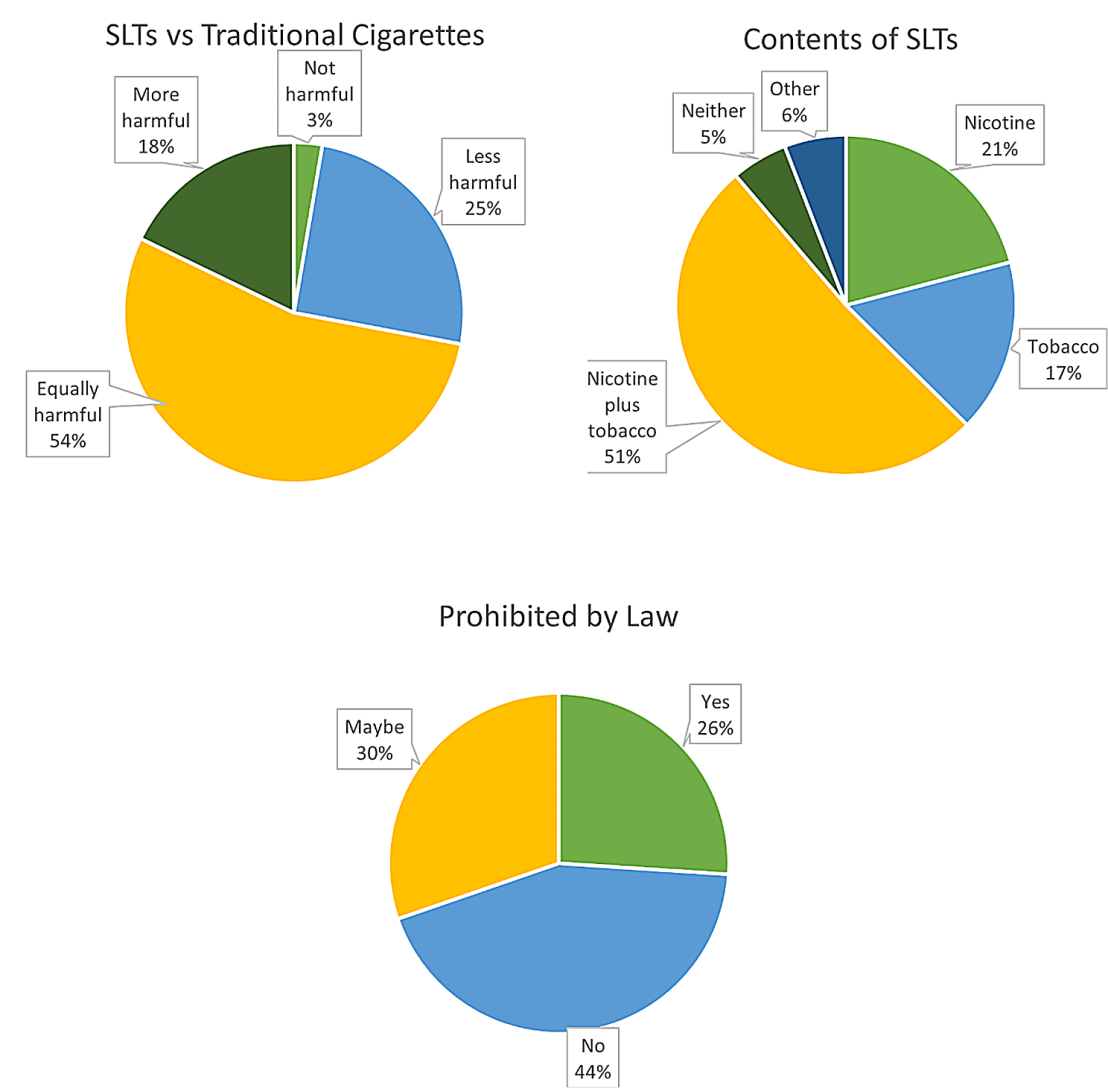
General knowledge regarding SLTs

Most participants (85.86%) demonstrated prior awareness of SLTs before the survey, selecting yes when asked if they had heard of SLT prior to this survey. When questioned about the contents of smokeless tobacco, 51.38% believed it contained both tobacco and nicotine, 20.92% believed it contained only nicotine, and 16.49% believed it contained only tobacco. Approximately 5% of participants held the view that neither substance was present. In terms of perceptions of harm, 54.23% believed that smokeless tobacco is equally harmful as traditional cigarettes, while 25.27% considered it less harmful, and 17.82% deemed it more harmful. A minor percentage (2.68%) perceived it to be not harmful at all. Notably, 43.68% believed these products were not prohibited by law, and 30.29% were uncertain about their legal status as displayed in Fig. 2.

**Fig. 3:**
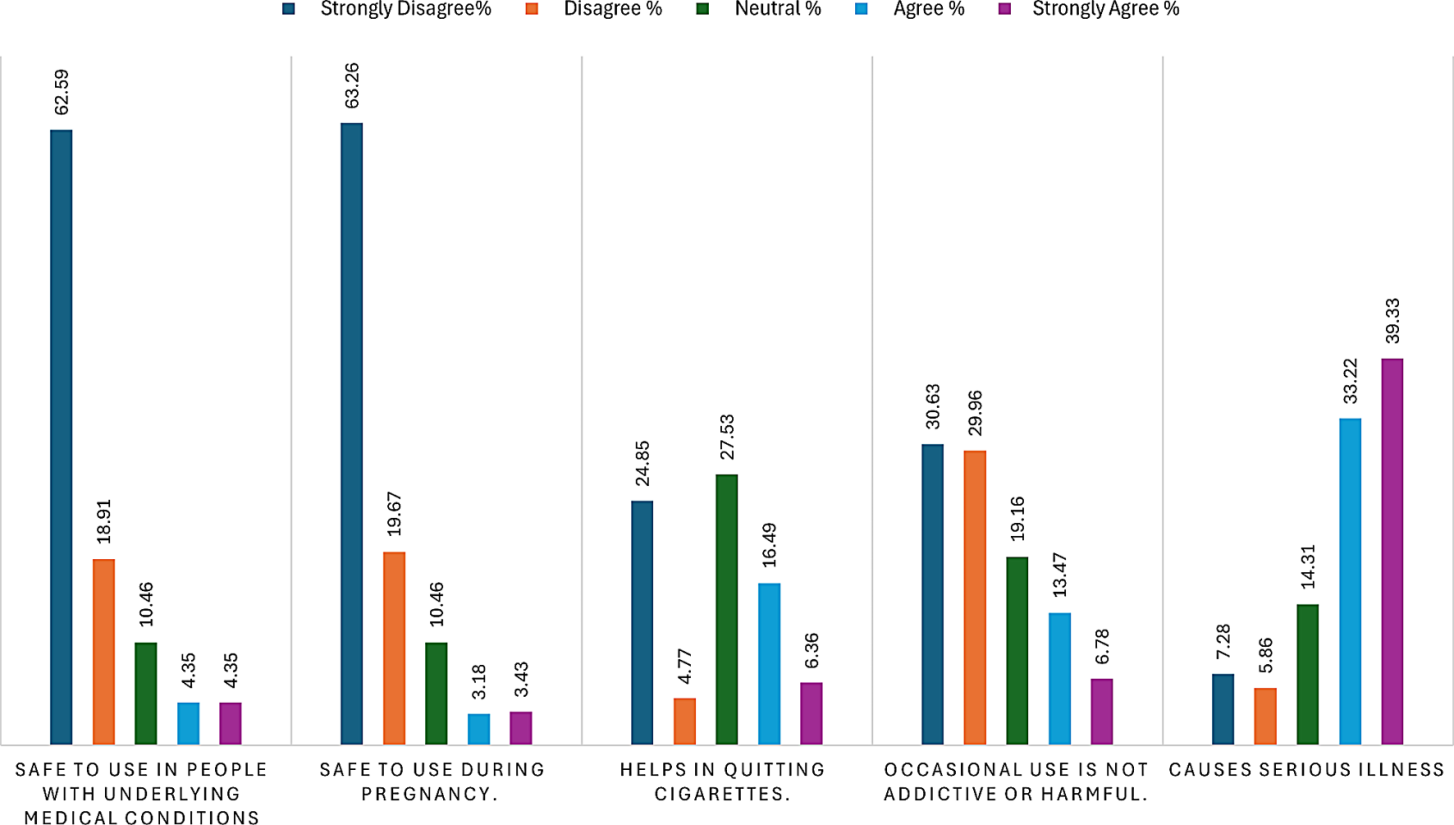
Health-associated knowledge regarding SLTs

Concerning health risks, 72.55% concurred that these products can lead to serious illnesses such as cancer and stroke. In addition, a majority disagreed with the notion that occasional use of these products is non-addictive or harmless, with 30.63% expressing strong disagreement. Meanwhile, 22.85% believed these products assist in quitting cigarettes, and 27.53% adopted a neutral stance on the matter. Finally, a substantial proportion, 63.26%, strongly disagreed that these products are safe during pregnancy, and 62.59% strongly disagreed that they are safe for individuals with underlying medical conditions such as heart disease, blood pressure, and diabetes as displayed in Fig. 3.

On linear regression analysis (Table 2) to determine significant factors affecting knowledge, females had greater odds to higher knowledge scores, respondents from Punjab had 33% lower odds of higher knowledge compared to respondents from Sindh. Higher monthly household income (1.8 times greater odds of the 100,000-500,000 group) and doctor visits (greater than 1) both were associated with greater odds of higher knowledge scores.

**Table 2:** Linear regression to determine factors affecting knowledge regarding SLTs

Variable	Odds Ratio	*P*-Value
**Age (years)**	1	0.295
**Sex**		
Female	1 (reference)	
Male	0.73	0.004*
**Level of Education**		
No formal education	1 (reference)	
< 5 years (Primary school)	5.52	0.607
5–10 years (Matric/ O level)	24.12	0.174
10–12 years (Intermediate/ A level)	25.37	0.115
12–14 years (bachelor’s degree)	17.82	0.137
> 14 years (Postgraduate degree)	17.74	0.146
**Marital Status**		
Unmarried	1 (reference)	
Married	0.92	0.557
Divorced	2.91	677
Widowed	2.34	0.533
**Province of Residence**		
Sindh	1 (reference)	
AJK	0.91	0.806
Balochistan	0.79	0.363
Gilgit Baltistan	2.64	0.233
Islamabad Capital Territory	0.84	0.534
KPK	1.02	0.888
Punjab	0.67	0.001*
**Monthly Household Income (Pakistani Rupees)**		
< 25,000	1 (reference)	
25,000–50,000	0.76	0.736
50,000-100,000	1.09	0.207
100,000-500,000	1.8	0.007*
> 500,000	1.45	0.159
**Number of Doctor Visits in Past 12 Months**		
0	1 (reference)	
1 or 2	0.97	0.03*
3 to 5	1.24	0.797
6 or more	0.93	0.094

Bold values denote significance

### Attitudes towards SLTs

**Fig. 4:**
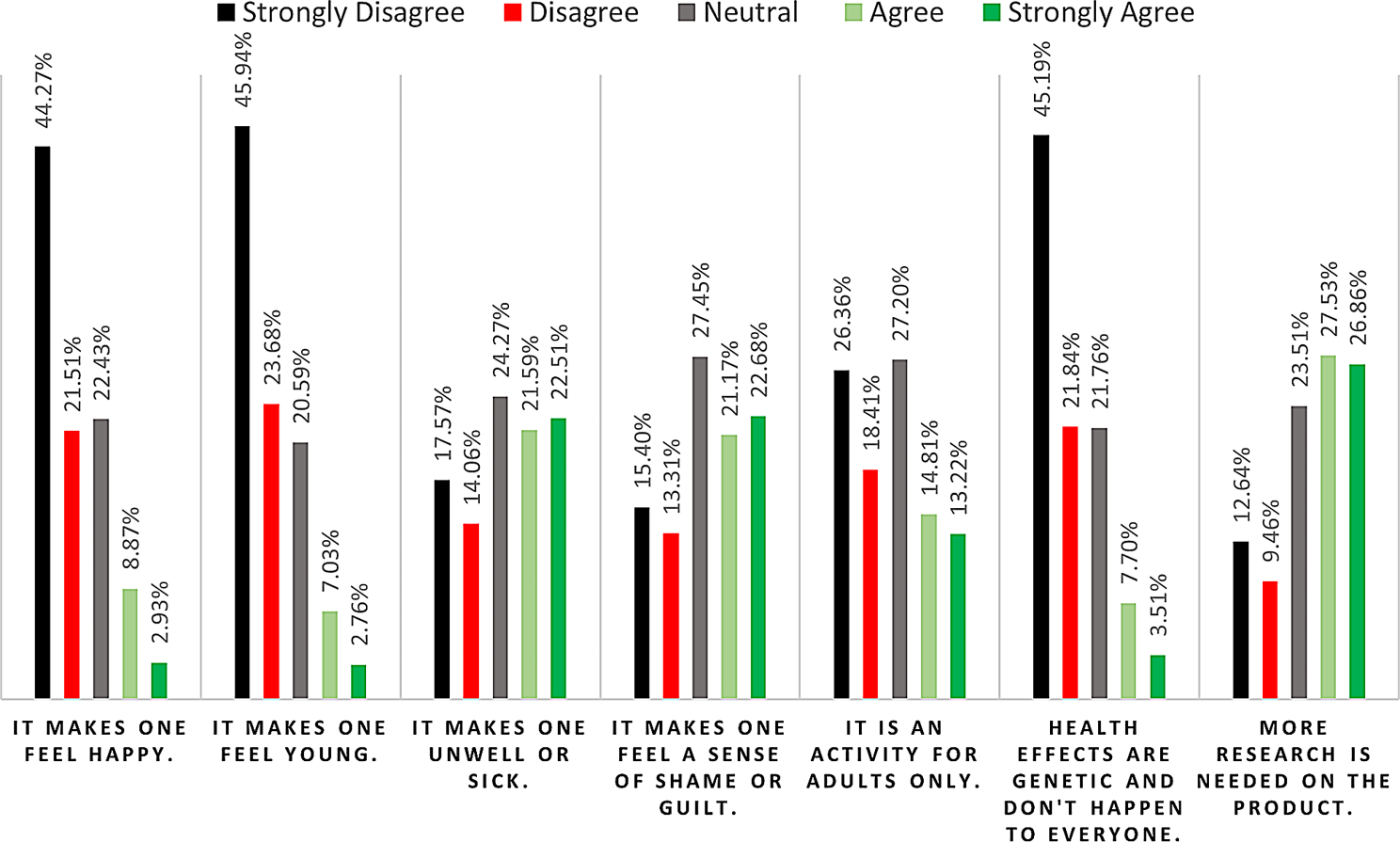
General attitudes towards SLTs

Concerning attitudes toward smokeless tobacco, a significant majority either disagreed or strongly disagreed that it makes one happy (65.78%) and feel young (69.62%). Conversely, participants agreed or strongly agreed that the use of these products could lead to feeling unwell or sick (44.10%) and evoke a sense of shame or guilt (43.85%). Interestingly, the majority also expressed disagreement or strong disagreement with the notion that these products are exclusive to adults (44.77%) and that the health effects are genetic and don’t happen to everyone (67.03%). Additionally, a substantial number of participants (54.39%) believed that further research on these products is required. These data are summarized in Fig. 4.

**Fig. 5:**
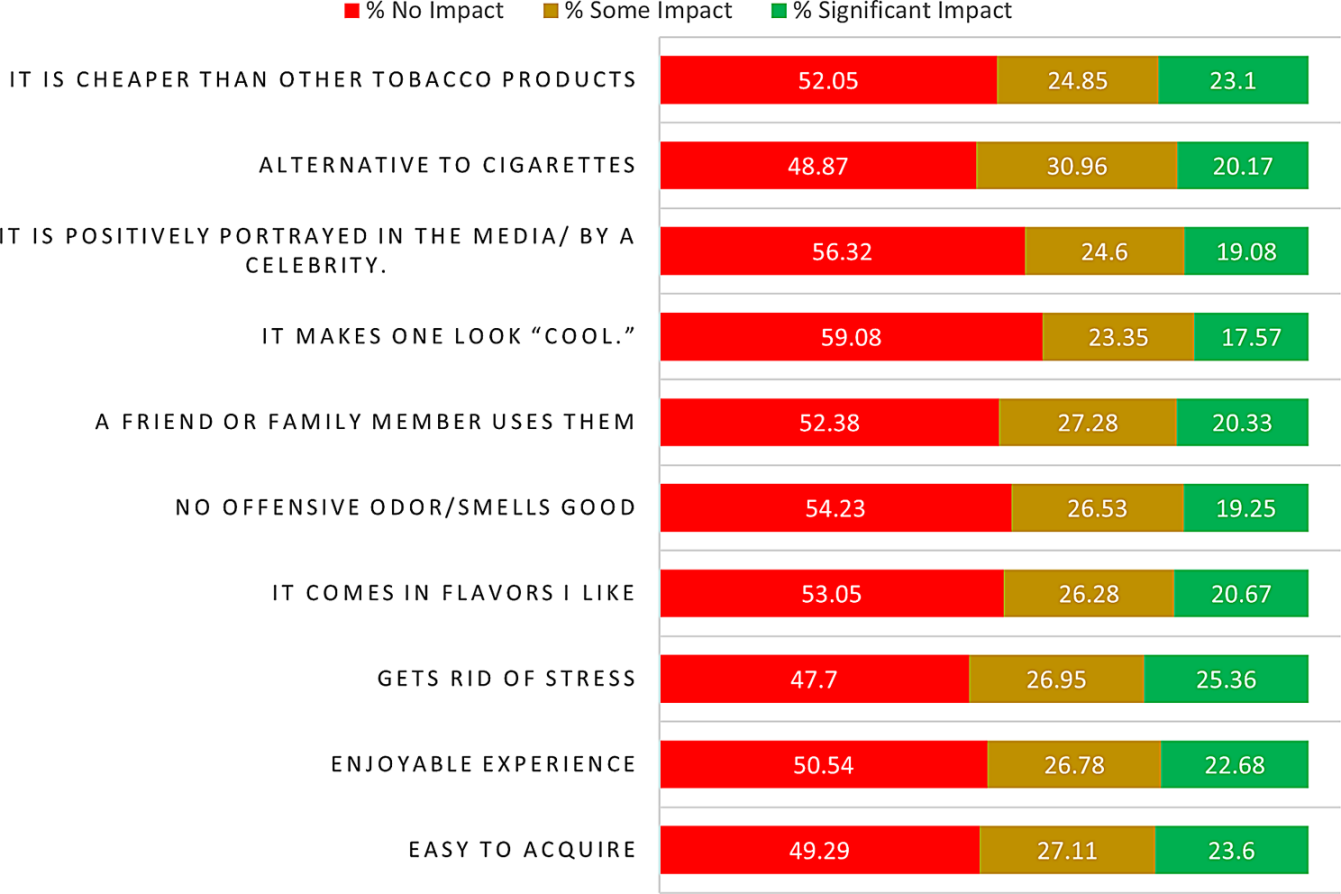
Factors encouraging the use of SLTs

We investigated the influence of various motivators on prompting individuals to consider or adopt smokeless tobacco products as shown in Fig. 5. Notably, the most substantial impact was attributed to the perception that these products aid in stress reduction, with 25.36% of participants acknowledging a significant impact. Furthermore, the pleasurable experience offered by these products had a significant impact on 22.68% of respondents, and the ease of acquisition played a significant impact on 23.60%. Motivations such as favorite flavors (46.95%) and the absence of offensive odors or pleasant smells (45.78%) were influential. Social factors were also relevant, as having friends or family members using these products (20.33%) and the perception of appearing “cool” (17.57%) held a significant impact. Positive media portrayals (19.08%) and the perception of these products as an alternative to cigarettes (20.17%) were additional contributors to motivation.

**Fig. 6:**
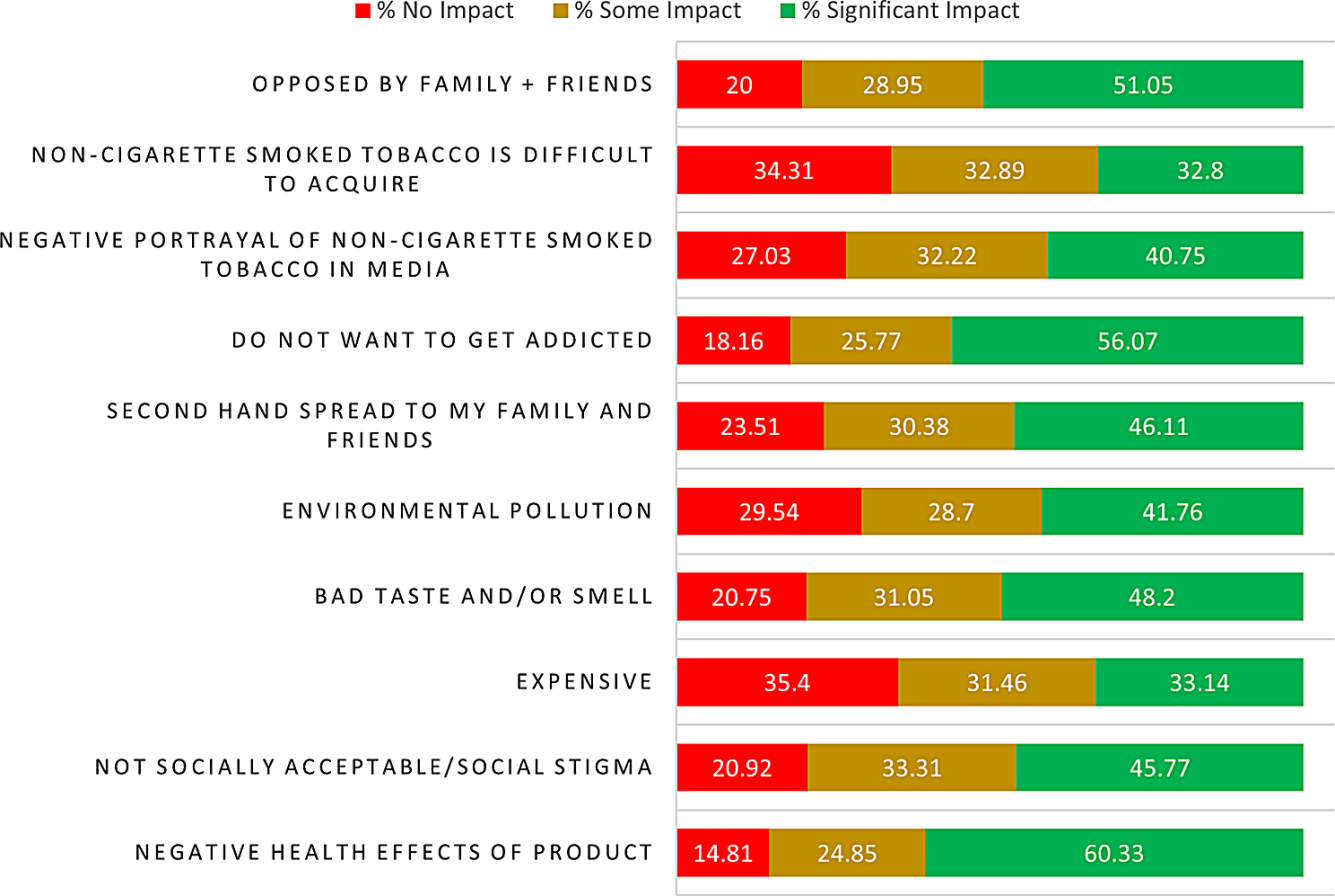
Factors deterring use of SLTs

An evaluation of various deterrents discouraging individuals from embracing smokeless tobacco products revealed notable impacts. Foremost among these deterrents was the concern about the negative health effects of the product, as reported by 60.33% of participants. Similarly, the social unacceptability or stigma associated with these products had a significant impact on 45.57% of respondents. 33.14% respondents claimed that the cost factor played a significant role in deterring them from using these products. Factors such as negative taste and smell (48.20%), environmental pollution (41.76%), and the potential spread of secondhand smoke to family and friends (46.11%) emerged as demotivators. Additionally, a significant percentage expressed that the fear of addiction (56.07%) and opposition from family members and friends (51.05%) had a significant impact in not pursuing them. These data have been displayed in Fig. 6.

### Usage of SLTs

In terms of usage, a total of 30.54% of respondents acknowledged having used smokeless tobacco products at least once, while the majority (69.46%) had not. Among past users of SLTs, 20.92% used it daily, while the majority used it less than daily (47.96%). In comparison, 31.95% of current users used SLTs daily while 35.50% used it less than daily. Concerning intentions to quit, 69.82% of current users expressed a desire to quit, while 30.18% indicated no plans to quit. These data have been summarized in Table 3.

**Table 3:** Usage of SLTs. *N* = 1195

Variable	*n* (%)
**Have you ever used any type of SLTs, even once?**	
Yes	365 (30.54)
No	830 (69.46)
Used SLTs at least once or currently using (*N* = 365)	
**Type of SLT or similar product used (more than one may apply per respondent)**	
Chewing tobacco, such as loose leaf, plug, twist roll	43 (11.78)
VELO/Oral Nicotine Pouches	88 (24.11)
Snuff/Naswar	20 (5.48)
Lozenges	13 (3.56)
Orbs	6 (1.64)
Sticks	7 (1.92)
Strips	7 (1.92)
Other	14 (3.84)
Past Users of SLTs (*N* = 196)	
**How often did you use smokeless tobacco products in the past?**	
Daily	41 (20.92)
Less than daily	94 (47.96)
Don’t know	61 (31.12)
Active Users of SLTs (*N* = 169)	
**How much do you currently spend on it per week? (PKR)**	
< 1000	45 (26.63)
1000–2000	25 (14.79)
2000–3000	10 (5.91)
> 3000	19 (11.24)
Don’t know	70 (41.42)
**How often do you use smokeless tobacco products currently?**	
Daily	54 (31.95)
Less than daily	60 (35.50)
Don’t know	55 (32.54)
**Do you plan on quitting?**	
Yes	118 (69.82)
No	51 (30.18)

Abbreviations used: PKR (Pakistani Rupees), SLT (Smokeless Tobacco)

On logistic regression (Table 4) to determine significant factors influencing the use of SLTs, the greatest odds of use were found with use as an alternative to cigarettes (OR 4.45) and secondly, due to its availability in a liked flavor (OR 2.27). Respondents who felt SLTs made them look cool had lesser odds of using them at least once (OR 0.58). No significant deterrents were found to influence SLT use.

**Table 4:** Logistic regression to determine factors influencing the use of SLTs at least once *Adjusted for age, gender, Province, ethnicity, Marital Status, Level of Education and Monthly Family Income

Variable	Odds Ratio	*P* -Value
**Motivators**		
Easy to acquire	0.68	0.181
Enjoyable experience	1.10	0.746
Gets rid of stress	0.92	0.800
Comes in flavors I like	2.27	0.008*
No offensive odor	1.33	0.317
A friend/family member uses	0.93	0.803
It makes one look cool	0.58	0.032*
Positively portrayed in media	0.77	0.326
Alternative to cigarettes	4.45	< 0.001*
Cheaper than others	1.04	0.891
**Deterrents**		
Negative health effects	0.93	0.816
Not socially acceptable	0.64	0.103
Expensive	0.88	0.601
Bad taste/smell	0.71	0.182
Environmental pollution	0.94	0.821
Second hand spread to family	0.82	0.485
Do not want to get addicted	1.18	0.576
Negative portrayal in media	0.82	0.478
Difficult to acquire	0.85	0.534
Opposed by family and friends	1.04	0.888

Bold values denote significance

## Discussion

Our study finds that the majority of the participants had heard of SLTs prior to the study and had a fair degree of knowledge regarding the product content and health effects, with negative health impact being a significant deterrent to use. It is to be noted that the laws pertaining to SLTs were debated among respondents and attitudes leaned towards the negative side. Motivating factors included as an alternative to cigarettes, the outlook of the drug on perceived popularity and widespread availability in flavors. The utilization of SLTs presents an escalating public health issue, with approximately 360 million individuals worldwide engaging in its consumption, predominantly concentrated in the South Asian region where over 90% of users reside [29]. Despite its well-understood association with various disease states, the prevalence of SLTs is steadily increasing within South Asian nations [13, 30, 31].

More than half the participants answered the composition of SLTs correctly, while at the same time a surprising 21% believed it contained only nicotine and 16.5% thought SLTs had tobacco only. The common forms of smokeless tobacco in Pakistan include Naswar, Chalia, Pan (betel with tobacco), and Gutka [32]. All of these compositions contain tobacco with varying degrees of nicotine based on regional preferences. A survey on rural Bangladeshi adolescents revealed that 32.5% of the participants did not know SLTs contain nicotine, and according to the Bangladesh Global Adult Tobacco Survey 2015, 58% of the non-users implicated that SLTs contain nicotine [33, 34]. Although lack of prior study examining the public’s knowledge of SLTs ingredients in Pakistan, hindered direct comparison, our findings are comparable to the available data.

Concerning health risks, 72.55% of our respondents concurred that these products can lead to serious illnesses such as cancer and stroke. One study from Karachi, Pakistan, revealed that 44% males and 37% females thought it was “bad for health”. 54% of our survey participants believed that SLTs are equally harmful, and 25% believed they are less harmful compared to traditional cigarettes [35]. This statistic is in accordance with a previous study conducted in Poland, which demonstrated that 72% of their respondents perceived SLTs to be as harmful and 25% to be less harmful than cigarettes [36]. Another survey from Bangladesh revealed that only one-quarter of their participants thought SLTs were less harmful than smoked tobacco [33]. The perceived severity of SLTs compared to traditional smoked cigarettes is low among our participants, thereby contributing to a higher likelihood of individuals choosing to use SLT [37].

Emotional associations with smokeless tobacco were also explored in detail in our study. Participants’ rejection of the notion that SLT use evokes a sense of feeling happy or young likely reflects an increasing awareness of the health risks associated with smokeless tobacco consumption [38, 39]. This shift in perception underscores societal attitudes, indicating a growing understanding of its adverse effects on health and well-being. Respondents associated a sense of guilt and unwellness with smokeless tobacco use likely stems from societal efforts to promote tobacco cessation, and it may contribute to individuals feeling morally conflicted and physically unwell when using smokeless tobacco products.

The majority of our respondents disagreed that smokeless tobacco products are exclusively for adults. In recent years, a shift from smoked tobacco products to SLTs is seen among the younger population in Western countries [40]. The drift towards smokeless tobacco in the South Asian younger population is also in the process. A recent study from India revealed that the majority of students initiating SLTs were in the 10–11 years age group, and 11% of the students enrolled in classes 9–11 were consuming SLTs [41]. The availability and accessibility of smokeless tobacco products, coupled with limited regulatory measures, contribute to their widespread use among younger populations [42].

Exploring the factors in encouraging the use of SLTs in our participants, we found that the perception of SLTs as a stress reliever, pleasurable experience after the use of these products, and ease of acquisition played a crucial role. A grounded theory study conducted in Iran, similarly, revealed that along with the addictive nature of SLTs, relaxation and euphoria were some of the prominent continuation factors [43].

Ease of acquisition and unregulated distribution play a pivotal role in the widespread prevalence of these products in the South Asian region [44, 45]. Naswar, a common form of SLT is widely available in 20 g packing costing 10 Pakistani rupees (US$0.04), which can be consumed several times a day [46, 47]. Social influences from friends and family members who use smokeless tobacco products can normalize and promote their usage among the population, particularly among young individuals who may be influenced by peer behavior [48]. Positive media representations of SLTs and their portrayal as an alternative to cigarettes can shape public perception, influencing individuals to perceive these products as attractive and potentially safer alternatives.

Pertinent factors in discouraging the use of SLTs in our respondents included negative health effects. Previous studies have demonstrated that the harmful health impacts of SLTs serve as the main deterrent [33]. The stigma attached to SLTs and the fear of addiction act as significant deterrents among our participants. The stigma stems from societal perceptions of these products as unappealing or undesirable, leading to fear of being judged, hence avoiding their use altogether. The fear of addiction underscores apprehensions about the long-term consequences of tobacco use, further discouraging its uptake.

A minority of respondents reported having used SLTs before, although higher than previous reports of 19–21% prevalence [49]. This is likely attributable to our sample having a greater degree of education and being primarily from the young adult age group. Previous studies have found associations with SLT use and age, with highest prevalence in the 30–39 age group and lowest in those with the highest level of education [50]. Future studies should be translated into regional languages of Sindhi, Pashto, Balochi and Seraiki and venture into rural areas of Pakistan and places where our web-based survey was unable to reach.

Of particular note in the patterns of use was that daily usage of SLTs had decreased from past to current users, signifying decreased initiative in procuring the product. A reason for this could be the warning given in tobacco packaging [51]. This impact can further be enhanced by ensuring the inclusion of all SLT kinds in the law and further increasing the degree of warning provided on packaging, a challenge the government continues to face [52]. This serves to show that interventions, if conducted in a proper manner have the potential to give results.

### Strengths

This study is first of its kind in this region to inquire on knowledge, attitudes and usage of SLTs in Pakistan and has the potential to pave the way for future research along with facilitating the development of policy. This study includes a new web based comprehensive questionnaire which was developed using elements from the Global Adult Tobacco Survey (GATs) and considerate reviewing was done to increase reliability of the questionnaire. An adequate sample size of 1195 participants were recruited, which increased the power of the statistical test.

### Limitations

The uneven representation throughout provinces is a crucial finding that highlights the need for more study to fully understand the various regional variations in the use of smokeless tobacco. Moreover, most of the study’s sample comprises persons with greater educational attainment, potentially skewing the findings and limiting their generalizability to the broader population. Additionally, as one of the pioneering investigations into SLT use in Pakistan with a substantial sample size, the study lacks comparative data from similar studies, hindering the ability to contextualize its findings. Our study focuses on younger university students who have a greater awareness of health risks and are more impacted by social stigma. This limits our ability to identify accurate deterrents for smokeless tobacco (SLT) use within this group and highlights the need for further research on factors influencing SLT use in a broader population. Future survey tools should also consider validity methods such as construct and convergent, which were not tested in the current study.

### Study recommendations and policy implication

SLTs have been in the tobacco market for a long-time making interventions to reduce use of public health priority. Public interventions should aim to reduce accessibility to the product while continuing to promote public awareness campaigns on the health risks of chewing tobacco. By making SLTs less inexpensive, greater taxes can discourage use. In order to restrict advertising and accessibility, it is also crucial to enforce strict marketing laws. In addition, with the emergence of branded forms of SLTs, monitoring of marketing is important to prevent early age exposure. Equally important is packaging, which can reduce attraction and warn consumers of potential hazards by requiring plain packaging with health warnings. Mandating plain packaging with prominent health warnings can further deter use. Campaigns directed by the government should encourage cessation support, and marketing efforts should be evidence-based and highlight health risks. By implementing these policy measures, Pakistan can advance its tobacco control efforts ultimately reducing the public health burden due to SLT use.

## Conclusions

In summary, our research contributes to a better understanding of SLTs in Pakistan. Our findings underscore the urgent need for targeted public health interventions to address misconceptions, deter use, and promote cessation efforts in the face of an escalating public health issue. SLTs have been within public use in the form of chewable tobacco for a long time, however the emergence of newer varieties requires policy intervention to regulate usage. By identifying key misconceptions, motivations, and deterrents, we provide valuable insights that can inform the development of targeted interventions aimed at reducing smokeless tobacco use and its associated health risks in the Pakistani population.

## Data Availability

Additional data is available on reasonable request from the corresponding author. Please note that all data has been provided in the manuscript.

## Abbreviations

SLT: Smokeless tobacco
STROBE: Strengthening the Reporting of Observational Studies in Epidemiology
AKUH: Aga Khan University Hospital’s
ERC: Ethical Review Committee
GATs: Global Adult Tobacco Survey
CVI: Content validity index
SPSS: Statistical Package for Social Sciences
IQR: Interquartile range
PKR: Pakistani Rupees
OR: Odds Ratio
AKU: Aga Khan University
